# Modernizing and designing evaluation frameworks for connected sensor technologies in medicine

**DOI:** 10.1038/s41746-020-0237-3

**Published:** 2020-03-13

**Authors:** Andrea Coravos, Megan Doerr, Jennifer Goldsack, Christine Manta, Mark Shervey, Beau Woods, William A. Wood

**Affiliations:** 1 Elektra Labs, Boston, MA USA; 2 Harvard-MIT Center for Regulatory Science, Boston, MA USA; 3 Digital Medicine Society (DiMe), Boston, MA USA; 4 Biohacking Village, Washington, DC USA; 5 Sage Bionetworks, Seattle, WA USA; 6 I Am The Cavalry, Washington, DC USA; 7 Atlantic Council, Washington, DC USA; 8 University of North Carolina Lineberger Comprehensive Cancer Center, Chapel Hill, NC USA

**Keywords:** Technology, Health policy

## Abstract

This manuscript is focused on the use of connected sensor technologies, including wearables and other biosensors, for a wide range of health services, such as collecting digital endpoints in clinical trials and remotely monitoring patients in clinical care. The adoption of these technologies poses five risks that currently exceed our abilities to evaluate and secure these products: (1) validation, (2) security practices, (3) data rights and governance, (4) utility and usability; and (5) economic feasibility. In this manuscript we conduct a landscape analysis of emerging evaluation frameworks developed to better manage these risks, broadly in digital health. We then propose a framework specifically for connected sensor technologies. We provide a pragmatic guide for how to put this evaluation framework into practice, taking lessons from concepts in drug and nutrition labels to craft a connected sensor technology label.

## Introduction

Over the past decade, the adoption of digital technologies in medicine—from electronic health records to wearable sensors—has occurred faster than the healthcare community’s ability to evaluate and secure these products^1–3^. The fundamental goal of any biomedical product evaluation is to assure that, in the intended context of use, the benefits of deploying the technology outweigh the potential risks to the participant/patient and the organization. As new technologies enter medicine and biomedical research, manufacturers, regulators, clinicians, and patients are relying upon existing regulatory and evaluation frameworks. For example, traditionally consumer-facing companies, such as Apple, are now approaching the US Food and Drug Administration (FDA) as they develop products for clinical settings^4^. However, the risks of deploying these new technologies are inadequately understood and are not protected by what are quickly becoming legacy evaluation frameworks.

Compared to legacy biomedical products, digital technologies have features that change the benefit-risk calculation; therefore, we must adapt evaluation frameworks. In this manuscript, we focus specifically on connected biometric monitoring technologies, which we will refer to as “connected sensor technologies”. Connected sensor technologies are digital medicine products that perform algorithmic processing of data captured by mobile sensors to generate measures of behavioral and/or physiological function. Examples of connected sensor technologies include smartwatches that measure activity, connected monitors that sit on top of mattresses to measure sleep, wireless arm cuffs that measure blood pressure, and microphones that capture vocal biomarkers signaling changes in brain health^5–7^. Notably, we intentionally use the phrase “connected sensor technology” or “connected product,” and we avoid the phrase “connected device” because “device” is an FDA term of art that refers specifically to cleared medical devices^8^.

In this paper, we first briefly discuss the benefits of connected sensor technologies and dive deeper into the risks these technologies present. Next, we outline the frameworks that are emerging across the industry to evaluate digital health, highlighting their strengths and shortcomings with reference to the connected sensor technologies industry. Finally, building on these emerging frameworks as a guide, we outline a practical guide for evaluating fit-for-purpose connected products across biomedical research and clinical care.

## Features of connected sensor technologies and their benefits and risks

Connected products are being rapidly adopted, with the number of wearables worldwide estimated to increase from 325 million in 2016 to 929 million by 2021^9^. The proliferation of wearables, ingestibles, and other connected sensors is making it easier than ever to collect high-quality behavioral and physiological data outside of the clinic^6^. Remotely collected data allow clinicians to discover sights that are more reflective of patients’ day-to-day experiences.

For drug developers, connected sensor technologies can improve efficacy^6^, increase inclusivity^10^, and lower the costs of conducting clinical trials^11^. For clinicians, these products can capture insights that are more reflective of patients’ day-to-day experiences, potentially resulting in major improvements in care delivery.

To capture these potential benefits, risk-benefit analyses are essential to ensure accurate measurement and patient safety in study protocols and clinical care. In the next section we highlight five dimensions that carry risks posed by connected sensor technologies: (1) validation, (2) security practices, (3) data rights and governance, (4) utility and usability; and (5) economic feasibility.

### Verification and validation

To determine the appropriateness of using a particular product, a short-cut question that often arises is whether the product is “validated” (e.g., “is this wearable clinically validated?”). Validation carries widely different meanings for different stakeholders. For instance, the pharmaceutical industry may use “validation” as a substitute for “GxP”, which is a generalized abbreviation for “good practice” quality guidelines and regulations. Examples of GxP are Good Clinical Practice (GCP), Good Manufacturing Practice (GMP), and Good Laboratory Practice (GLP). GxP compliance is a set of quality system of management controls which have been developed over the years with and for stakeholders (e.g., clinical trialists, manufacturers, laboratories), and codified into current regulatory regimes.

For others, validation may be more akin to “analytical or clinical validation,” referring to the quality of the measurement coming from the sensor and algorithms that compose the connected sensor technology. Others may also bundle “validation” with “verification and validation” (V&V), quality management procedures that ensure that the system or product meets specifications and that it fulfills its intended purpose (e.g., “software V&V”). Over time, evaluation frameworks that are developed for connected sensor technologies will likely be codified into revised GxP and related quality management systems; however, to develop good practices, the underlying principles must first be established.

To account for the unique hardware, software, and algorithmic properties of connected biometric monitoring technologies (BioMeTs), we recommend the three-stage process of verification, analytical validation and clinical validation (V3) proposed by Goldsack, Coravos, Bakker et al.^12^. In this framework:

- **Verification** evaluates and demonstrates the performance of a sensor technology within a BioMeT, and the sample-level data it generates, against a pre-specified set of criteria.
- **Analytical validation** evaluates the performance of the algorithm, and the ability of this component of the BioMeT to measure, detect, or predict physiological or behavioral metrics.
- **Clinical validation** evaluates whether a BioMeT acceptably identifies, measures, or predicts a meaningful clinical, biological, physical, functional state, or experience in the specified population and context of use.

A strong V3 process serves as the foundational evidence base around the accuracy, reliability, and appropriateness of the data and results from connected sensor technologies. Nonetheless, conducting a successful V3 process is challenging for a number of reasons. First, most sensor-based products are comprised of a modular stack of hardware and software components, from sensors to signal processing to algorithms^6^. Each component may be built by a different company, each of which contribute to the product’s overall V3 results. Second, a change earlier in the data supply chain (e.g., at the signal-processing algorithm in the sensor) may alter the data inputs for an algorithm high-up the chain, which may result in an entirely new V3 valuation^12^. Put another way, it is challenging to evaluate a connected sensor technology’s data supply chain, the data flow and data provenance for information generated from hardware, sensors, software, and algorithms. Indeed, it requires modifications to the V&V process for wet-lab tests or clinical outcome assessments (COAs) like electronic patient reported outcomes (ePROs).

### Security

By definition, connected sensor technologies transfer data over the internet, which introduces immediate risks when deploying these products, because an actor could attack and access the product remotely and often in near-real time. This second dimension on Security risk evaluates unauthorized uses of data and results; the following section on Data Rights and Governance evaluates authorized uses of data and results. Cybersecurity involves protecting internet-connected systems, data, and networks from unauthorized access and attacks, including human error (e.g., the loss of a company’s unencrypted laptop). Notably, some data and system access may be authorized (or perhaps “not forbidden”), though unwelcome or undisclosed to the patient or other stakeholders. This type of access will also be covered in the next section. Although the security of a system cannot be guaranteed, quality design and execution can decrease the risk of harm from code flaws, configuration weaknesses, or other issues. A product’s security risk will need to be continuously re-assessed as new technologies and attack methods become available (e.g., advances in quantum technologies and corresponding quantum-resistant encryption standards).

### Data rights and governance

When we consider data rights, we prefer to refer to governance rather than privacy, because we believe it’s more important to empower individuals to choose how to share their date—their rights and governance—rather than defaulting to privacy (e.g., a patient with a rare disease may want more freedom rather than barriers to share her data and results with relevant parties).

Over the past year, many popular tech companies have come under greater scrutiny for how they choose to share data with third-parties. For instance, the Cambridge Analytica incident with Facebook was not an unauthorized use or attack on the Facebook network (e.g., it was not a security incident). Aggregation of data in the ways utilized by Cambridge Analytica was part of Facebook’s feature set, though many have argued this feature was not thoroughly disclosed to all parties. Examples of wide-spread data sharing with inadequate disclosure is also seen in health tech products. Huckvale et al found in a cross-sectional study of 36 top-ranked apps for smoking cessation and depression in public app stores, “29 transmitted data to services provided by Facebook or Google, but only 12 accurately disclosed this in a privacy policy”^13^.

It is important to note that the regulatory environment is far from established when it comes to governing “digital specimens” (e.g., the data generated from connected sensor technologies). With respect to regulation, the FDA has oversight for digital specimen-collecting technologies, like wearables, when they are classified as a medical device. However, due to the narrow definition of device and the revisions with the 21st Century Cures Act, many connected sensor technologies fall outside of the FDA’s purview^14^. These narrow frames leave oversight of connected sensor technology functionality and health claims primarily to the Federal Trade Commission, which policies unfair and deceptive trade practices, including enforcing rules against false or misleading advertising^15^. In the United States, other agencies like National Institute of Standards and Technology (NIST), Federal Communications Commission (FCC) and Office of the National Coordinator for Health Information Technology (ONC) may each have oversight of components of connected sensor technologies, but no regulator has full responsibility for digital specimens. Given this ambiguous regulatory landscape, end-user license agreements (EULAs) for sensors with downloadable software (e.g., app), terms of service (ToS) for sensors themselves, and privacy policies (PP) have become the de-facto agreements that to retain rights in software and to create rights to monitor, aggregate, and share users’ digital biospecimens (see Box 1)^15^.

Box 1. Data rights disclosures: EULAs, ToS and PP intended use cases **Privacy policies (PP)** disclose the terms for collection and use of the app/website user’s personal information.

**Terms of service (ToS)** disclose the rules and requirements of website and/or app use, for example, copyright, allowed uses, and the definition of abusive use.

**End-user license agreements (EULAs)** are a form of intellectual property licensing that tell people who have purchased software if/how many times they can copy the software and how they can or cannot use those copies.

### Usability and utility

Commonly, concepts around verification and validation are confused with “clinical utility”. Clinical utility, defined as the process of evaluating whether the product improves health outcomes or provides useful information about diagnosis, treatment, management, or prevention of a disease, is also necessary to determine fit-for-purpose^16^. Clinical utility is typically evaluated by a process of user experience testing. It is common to define a product’s “usefulness” as usability plus utility^17^. Put simply, “utility” is whether a product has the features that users need, and “usability” is how easy and pleasant those features are to use^18^. If a product has high utility, people are often willing to accept lower usability thresholds. Connected sensor technologies require a web of participants to function successfully across the patient, the clinic/site, and the software-integration. Therefore the usability and utility has to be considered across multiple roles, including but not limited to the individual patient, the clinician/researcher, software engineer and data scientists who are using the product. For instance, the product must be easily understandable for the clinician or researcher to explain why and how to use it, for the patient to put it on and activate the product consistently during the observation period, and for the engineers and data scientists to ingest and analyze the data (e.g., if the product has poorly documented communication protocols or is hard to download/upload data, then the engineering team will struggle to make sense of the data).

### Economic feasibility

Compared to drugs, which often use a per-use pricing structure, or a traditional medical device, with a one-time purchase price, connected sensor technologies typically deploy a different business model, such as a subscription or long-term fees around data storage and analysis. These software-as-a-service fees may also cover additional software development, such as developing and shipping cybersecurity patches for software updates. Given that connected sensor technologies may shift their pricing and business models over their lifetime, it may be difficult to calculate a connected sensor technology’s economic feasibility, defined as the degree to which a product’s economic advantages are greater than its costs^19^.

## Emerging digital health evaluation frameworks

**Fig. 1:**
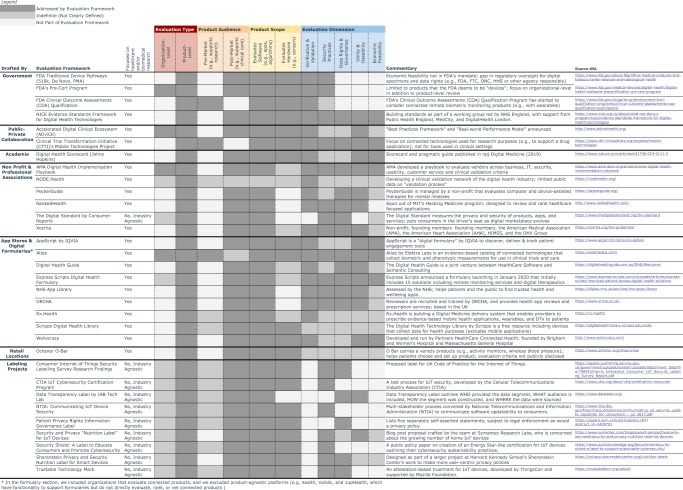
Current Evaluation Frameworks for Connected Sensor Technologies. This figure illustrates many of the known evaluation frameworks for connected technologies, categorized by source, type, audience, scope and dimension. References are provided.

Fortunately, many stakeholders have already started to revise and create improved evaluation frameworks to better understand digital health benefits and risks. In response to new digital technologies flooding the market, the FDA has issued a number of guidances to “encourage innovation and enable efficient and modern regulatory oversight”^20^, and multiple organizations have proposed improved standards and tools to better understand a technology’s risk-benefit analysis (Fig. 1).

Within these emerging frameworks, there are a few themes:

1. **Product versus organizational-level evaluations**. First, emerging frameworks contemplate whether the technology should be evaluated at the product-level, organization-level or both. Historically evaluations focused on the product, not on the organization or manufacturer (e.g., FDA judges the quality of a specific drug, and not Pfizer overall). With connected sensor technologies, the broader system needs to be taken into context, because a hardware or software change in one component (e.g., an update to the signal processing algorithm) can impact the system overall. Additionally, because software updates can occur frequently—in some instances multiple times per day—regulating these changes can require a new framework to manage risk. In software development the culture and processes at the organizational-level impact multiple products at once. Organizational-level views can also be better than product-level views when considering data rights and governance, as privacy policies and EULAs are often structured at the organizational-level rather than individual product-level. Therefore, some evaluation frameworks have shifted to consider this organizational view such as the FDA Precertification (Pre-Cert) Program, which evaluates the quality of the organization overall and then provides a “streamlined” review pathway for pre-certified organizations^21^.
2. **Research versus clinical care settings**. Second, the same connected sensor technology may be used in either a research setting (e.g., to collect digital endpoint data to support a drug application) or in a clinical setting (e.g., to remotely monitor a patient’s quality of life). An optimal evaluation framework should likely have the same base evaluation for the quality of the connected sensor, and afterwards, adapt the evaluation for different requirements in a clinical and research setting, respectively. This issue is exacerbated when regulatory requirements vary across connected technologies (i.e., some are regulated as medical “devices”, and others are not). The distinction between which digital health technologies are regulated and which are not is still evolving^22^, and a gray area can be dangerous for public health. Take for instance the controversies surrounding JUUL, a technology that was not well understood when first deployed into the market and now is facing greater regulatory scrutiny^23^. In a fast-paced technology world, it’s not only the responsibility of regulators to develop new evaluation models. With additional forethought, we do not have to wait for public crises to enact thoughtful oversight. It is the responsibility of all the parties involved to work towards understanding safe and effective products.
3. **Evaluation scope: software versus hardware review**. Given the modularity of connected sensors (e.g., a hardware component, sensors, signal processing algorithms, and apps to display the data), some emerging frameworks look at the whole set of components and others conduct a software-only review. Additionally, this modularity split is also showing up in a regulatory context as the FDA has introduced the concept of a “software as a medical device” (SaMD), which is “software intended to be used for one or more medical purposes that perform these purposes without being part of a hardware medical device”^24^.
4. **Comprehensiveness**. The final theme is that some of the emerging frameworks review all five of the risks posed by connected sensor technologies (validation, security, data rights and governance, utility and usability, and economic feasibility), and others only look at a subset. In the following section, we build off the lessons from the emerging frameworks and propose a pragmatic evaluation criteria to consider when deploying connected sensor technologies in research or clinical care.

## Building an evaluation framework for connected sensor technologies

**Fig. 2:**
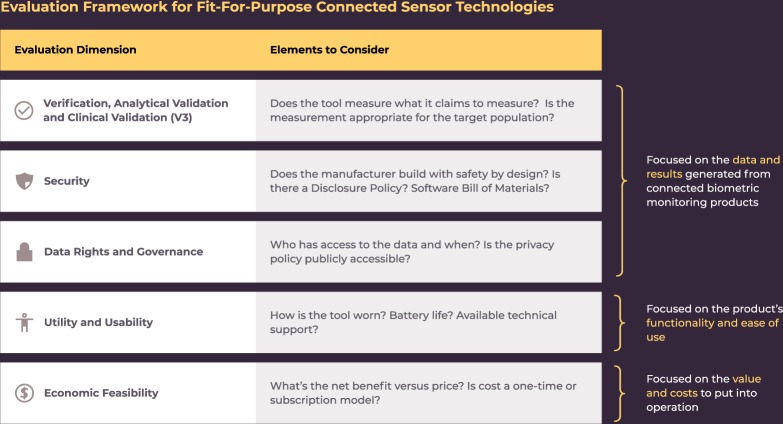
Proposed Evaluation Framework for Fit-For-Purpose Connected Sensor Technologies. The proposed framework describes the key dimensions to be considered when evaluating connected sensor technologies. Dimensions can be grouped into broader categories of data and results; functionality and ease of use; and value and costs.

Building on the existing frameworks, we propose a working evaluation framework for connected sensor technologies that reflects the five types of risks identified above (Fig. 2). We constructed this framework using the following principles:

1. **Evaluation criteria should be objective, observable, and verifiable** (see Box 2). Objective criteria are clearly and reliably measurable. Observable criteria can be checked independently, without special or privileged access. Verifiable criteria can be demonstrated or refuted with empirical data.
2. **Evaluations need context**. “What is the best drug?” or “What is the best food?” are meaningless questions without additional context (e.g., does the person need less sugar in her diet? Or more protein?). Similarly, “what is the best heart-rate monitor?” is an empty question without a clearly articulated context.
3. **Evaluations should be multidimensional** (e.g., avoid a single metric “score”). While scoring a food by a single metric such as total calories can be helpful, calorie count in itself is not a way to construct an overall healthy diet. Similarly, we argue that compressing an evaluation of a Fitbit versus an Apple Watch into a single overall score lacks meaningful nuance.
4. **Evaluation components can have required minimum thresholds and optional features that enhance the desirability of the product**. Required thresholds of each component may depend on the risk level and context of use.

We propose a systematic and standardized approach to evaluate whether connected biometric monitoring technologies are “fit-for-purpose” across five dimensions:

1. Verification, analytical validation, and clinical validation (V3)^12^;
2. Security practices
3. Data rights and governance
4. Utility and usability; and
5. Economic feasibility

The first three dimensions evaluate the data and subsequent results generated by connected biometric monitoring products. The fourth dimension, Utility and Usability, evaluates the ease of implementation and adoption of the product, and the last dimension, Economic Feasibility, evaluates the economic feasibility of adoption.

Notably, excellence in one dimension does not necessarily imply excellence in another. Indeed, significant deficiencies in any one dimension may lead to problems when using a connected sensor technology in research or practice. Thus, we propose a framework to simplify the evaluation process of connected sensor technologies for their intended uses.

Box 2. Objective, observable and verifiable evaluation criteria **Objective** That it can be agreed whether the capability is in place - often this leads to binary proofs - it either is or is not, there are no degrees.

**Observable** That an independent person can know whether the capability exists, without special or privileged access. This characteristic gives public scrutability to the capability.

**Verifiable** That a capability can be demonstrated or refuted with empirical data.

Source: Adapted from comments on NIST considerations^51^.

## Key evaluation criteria and metrics

### Verification, Analytical Validation, and Clinical Validation (V3)

The following documentation is necessary to determine net benefit using V3 principles:

- **Verification** can look like performance specifications for integrated hardware, overviews of software system tests, or output data specifications^12^. Often this information is on the manufacturers’ website and not as a peer-reviewed journal article.
- **Analytical validation** may look like studies that follow Good Clinical Practice (GCP) requirements, and could show up as a regulatory submission (e.g., 510k), white paper, or peer-reviewed journal article.
- **Clinical validation** may look like a clinical study report (CSR), regulatory submission, journal paper or published conference proceedings^12^.

Of all forms of documentation, those that make available complete data sets for external review should be weighted most heavily, particularly when machine learning algorithms are used^25^. Emerging standards exist for the assembly and documentation of datasets^26^ and the visualization of underlying data quality^27^. There is also a need for a living database of published studies with these types of data, as efficiently gathering data across the medical literature in each of these areas can be challenging for end-users. The Clinical Trials Transformation Initiative’s (CTTI’s) Interactive Database of Feasibility Studies for Mobile Clinical Trials is a useful and welcome start^28^. The Digital Medicine Society (DiMe)’s crowdsourced library is another useful resource that lists connected sensor technologies that collected data used to derive digital endpoints for industry-sponsored studies of new medical products or new applications of existing medical products^29,30^. More work needs to be done to ensure reproducibility of machine learning algorithms in healthcare^31^, and whether or not publishing either the algorithms or datasets for an independent external review^32^ would be a constructive way to increase reproducibility and decrease bias^1^.

When evaluating V3 for a connected sensor technology, the product should have documentation for each of the three stages. The documentation should align with the intended patient population and context of use. Measures that have been clinically validated in one group of patients cannot be assumed to be valid in another group in which patient or environment characteristics may affect measure performance (e.g., gait in Parkinson’s population may be evaluated differently than in a population with Multiple Sclerosis). As a desired threshold, the measure should be evaluated and published in multiple populations and data sets. Regulatory decisions (e.g., from FDA or EMA) can be distracting or misleading when evaluating a product. For instance, a connected sensor technology’s 510(k) FDA clearance as a device for clinical practice has no impact on whether it would be a suitable product for a drug clinical trial. All the decision indicates is that an external body has reviewed the manufacturer’s marketing claims.

We recognize that many connected sensor technologies may not meet the minimum threshold for verification and validation. For such technologies, we recommend identifying where along the data supply chain the product is missing documentation, and then conducting and/or soliciting research to complete the V3 process.

**Sample threshold criteria for the V3 process**

- **Minimum threshold or pass/fail:** a proposal or initial white paper for how the product plans to run its V3 process (e.g., what’s been completed and where are the gaps).
- **Desired threshold:** white paper or equivalent data describing elements of the V3 process; depending on the intended use (unless an analytical or clinical validation study is planned), published V3 data within the context of use.
- **High quality:** well-documented V3 specifications; analytic and clinical validation data published in multiple populations and data sets.

### Security

The next two dimensions of the evaluation framework, Cybersecurity and Data Rights and Governance, address unauthorized and authorized access to data and systems. Although all connected sensor technologies will eventually fail, not all failures have to cause harm. In 2016, a group of security researchers published a set of principles for creating more resilient and secure systems. These principles, encapsulated as the Hippocratic Oath For Connected Medical Devices, include designing the product with cyber safety in mind, ensuring third-party collaboration, capturing evidence to improve safety investigations, resilience and containment, and an emphasis on cyber safety updates and patching^33^. All of these five principles were incorporated into the FDA’s pre- and post-market guidances for cybersecurity of connected medical devices^34,35^.

When evaluating a product’s cybersecurity considerations, it is critical to ensure the assessment is risk-based and the context of use is considered. There are many organizations that will conduct customized risk-based security assessments. Or, an organization may earn a certification, like HITRUST^36^.

When considering the cybersecurity metrics, we propose a mix of passed assessments and certifications as well as process-related objective metrics. For technologies connected to the internet, the question isn’t “if” the product will be compromised, but “when.” Because of this, we focus on process metrics around identifying vulnerabilities and successfully patching them^33^:

**Sample threshold criteria for the cybersecurity considerations (**see Box 3**)**

- **Minimum threshold or pass/fail:** the connected sensor technology manufacturer has a publicly accessible Coordinated Vulnerability Disclosure (CVD) policy and publishes details about updates, including security vulnerabilities addressed.
- **Desired threshold:** the connected sensor technology manufacturer maintains a software bill of materials (SBOM).
- **High quality:** the organization keeps track of patch uptake rate and makes its SBOM available to customers and regulators.

Box 3. Cybersecurity considerations Evaluation criteria considerations

**Does the company have a Coordinated Vulnerability Disclosure (CVD) Policy and what does it contain?** A Coordinated Disclosure (CVD) Policy outlines the process, rules of engagement, and expectations for security researchers (or others) who find and report potential security issues, in good faith^52^. When financial incentives are offered to individuals for reporting issues, this may be called a bug bounty program^53^.

**Does the organization publish its security support lifetime and issue secure, prompt, and agile software updates once security issues are discovered?** Software updates can correct and mitigate potential security and safety issues much more effectively and efficiently than hardware replacement. Transparency and clear communication increases update participation rates, protecting more patients. Sharing security documentation, such as minimum supported lifetimes and known Common Vulnerabilities and Exposures (CVEs) allows users of connected sensor technologies to make more informed decisions about purchasing and use.

**Does the organization track and share a Software Bill of Materials (SBOM)?** An SBOM list of components in a piece of software is analogous to a list of ingredients on food packaging^54^. Given that nearly all software products today are comprised of third-party components, an SBOM makes it easier to identify and address potential security risks from software component vulnerabilities.

### Data rights and governance

The third dimension addresses issues related to authorized (or “not unauthorized”) access to data and systems. Given the ambiguity in the regulatory landscape and the lack of clear regulatory oversight of this risk area, we focus on the individual product policies. As a result, the metrics we recommend evaluating for connected sensor technologies are related to EULAs/ToS and PPs. Sage Bionetworks, a nonprofit organization that promotes open science and patient engagement in the research process, has pioneered a number of open toolkits for informed consent and privacy best practices that we have drawn from in this example^37,38^.

**Sample threshold criteria for data rights and governance considerations (**see Box 4**)**

- **Minimum threshold or pass/fail:** EULA/ToS and PP exist, are comprehensive, and are publically accessible online.
- **Desired threshold:** Documents are comprehensible by broad audiences, as well as accessibility compliance (e.g., 508 Compliant) for people with disabilities^39^.
- **High quality:**
  - ⚬ EULA/ToS does not contain exculpatory language.
  - ⚬ Users can opt-in or opt-out of third party transfer/use of their data.
  - ⚬ The rights of users are not changed even in the case of a change in ownership of the connected technology/sensor manufacturer.

Box 4. Data rights and governance considerations Evaluation criteria considerations

**Does the device have EULA/ToS and PP?** EULAs are traditionally associated with software that is downloaded by the user; ToS are associated with devices that do not require additional download of software. All sensor/software/devices should additionally have privacy policies.

**Are these policy documents comprehensive?** EULA and ToS: are the limits of use and liability* disclosed? Are termination of access, transfer of holdings, and the policy for changes/updates to terms described?

PP: Is what digital specimens will be collected, how they will be used (including by third parties), and what recourse the user has regarding their digital specimen described?

**Are these documents physically accessible?** Documents should be publicly accessible online.

**Is the information contained in them comprehensible by broad audiences?** Is documentation written at or below the 5th grade reading level or are plain language summaries provided? Is documentation 508 compliant^55^?

*It is important that researchers understand the limits of liability disclosed by the device/sensor manufacturer and ensure that these do not constitute exculpatory language per FDA/OHRP guidance^56^.

### Utility and usability considerations at the participant, site, and software integration levels

In this dimension, we focus on utility and usability from three perspectives: the patient, the site, and the software-integration level.

For the person wearing the connected sensor technology there are many practical considerations. This includes the form factor (e.g., how is a wearable sensor worn, how does it feel, what does it look like), battery life, water resistance, and materials used in its construction (e.g., does it cause a rash or irritation?) Availability of technical support, features of associated software that display sensor-generated data, and how engaging the product is can affect the overall participant experience. If the product is too difficult for the patient to use (i.e., low patient usability), data may not be suitable for analysis because of high rates of data missingness or participant dropout.

For the clinical site or practice that must integrate the connected sensor technology into a trial or routine care, workflow considerations are important. For example, sites should consider statistical analysis and how the data from the connected sensor technologies are aggregated and made available for reporting (e.g., administrative dashboards, or EHR displays if applicable). Workflow issues might include how participants are trained to use the connected sensor technology; what level of site monitoring or support is needed as the data are obtained; what clinical actions (if any) are needed in response to the data and how thresholds or alerts to guide these actions are designed; and what type of site-level technical support is needed to ensure that the technology functions appropriately. If not adequately considered, the site may be unable to obtain health measurements or deliver interventions as desired, reducing the effectiveness and impact of the technology^40^.

By “software-integration perspective,” we refer to considerations related to the integration of a connected sensor technology with software necessary for the transfer, visualization, and analysis of the connected data. This may include the use of standard data models (e.g., FHIR or OMOP Common Data Model) and open application programming interfaces (APIs) to facilitate ingestion and analysis of digital specimens.

Finally, usefulness under ideal circumstances is different for particular sub-populations. For example, a connected sensor technology may perform well from a participant and site perspective when the population is a group of high-functioning, healthy volunteers and the environment is a highly equipped and controlled digital laboratory within a sponsor organization. The same technology may perform less well when the population is a group of older, multimorbid patients with life-limiting illnesses and low physical function, and when the environment is a busy medical center participating in a clinical study, or a “real world” direct to participant approach as part of a decentralized trial^10^.

When evaluating a product, we recommend that the connected sensor technology have data on its utility and usability in healthy volunteers, published in a related white paper or equivalent. As a desired threshold, we look for peer-reviewed evidence of the technology in healthy volunteers and patients in trials or practice.

Additional value is added if published data match the intended use of the technology, in the intended target population, for the intended duration of data collection, with low rates of data missingness, and with ease of data aggregation and analysis. Further value is added based on the level of published or unpublished detail available for each of the above that can guide those applying the connected sensor technology to the intended population. Lastly, an indirect criteria for usability of a connected sensor technology could be the extent to which it is currently used in research and practice. Thus, further value is added based on the extent to which it is being used in clinical trials, grant applications, and real-world settings.

**Sample threshold criteria for utility and usability considerations**

- **Minimum threshold or pass/fail:** white paper or equivalent data about the connected sensor technology in healthy volunteers.
- **Desired threshold:** peer-reviewed publication about the use of the connected sensor technology in patients.
- **High quality**: matching of feasibility data to intended context of use; high adherence/low data missingness; ease of data aggregation; detail to provide guidance; past and current use in research and practice.

### Economic feasibility

When considering economic feasibility, it is important to calculate both the advantages and costs when using the product. The advantages often will come from improved quality and outcomes from using the product. The costs will depend on the product’s pricing model and expected changes over time (e.g., to cover long-term data storage, analysis, and security updates). When conducting an economic feasibility analysis, the feasibility perspectives from an insurance company, clinician and patient would likely differ, and therefore it is also important to consider all of the potential stakeholders and the values of those participating in the system. One stakeholder’s advantage may be another stakeholder’s cost. Economic feasibility calculations for a system are often complex as many of the advantages do not always have financial metrics associated and the lifetime costs can be difficult to estimate.

## Operationalizing and deploying a connected sensor technology evaluation framework

An evaluation framework is an intellectual exercise until it is put into practice. In this section, we outline several pathways for operationalizing the framework we describe in this manuscript. We anticipate roles for regulators such as FDA, standards bodies like Xcertia and IEEE, and those who develop GxP processes for connected sensor technologies. We also need to have better communication infrastructure to make information more accessible to the stakeholders who evaluate and deploy these technologies, the patients, doctors, software engineers and so on.

The technique of labeling has been deployed as a communication tool for complex products in healthcare. Prescription drug labels communicate essential, science-based information to healthcare professionals. Nutrition labels communicate essential, easy-to-read information for consumers managing their diets. Labels are critical pieces of infrastructure that provide transparency and clarity for life-critical products. Efforts to improve Internet of Things (IoT) security transparency has the attention of the multiple government entities. In the United States Congress, the Cyber Shield Act has been proposed, which includes labeling elements to help consumers review the security features of devices they’re buying^41^; the National Telecommunications and Information Administration (NTIA) is exploring transparency tools such as product packaging labels and consumer-facing websites^42^; the United Kingdom government has developed labeling proposals and researched consumer perceptions^43^. Outside of governments, several more security and data-rights focused labels have been proposed such as Privacy and Security Nutrition Label for Smart Devices^44^, Symantec’s IoT label^45^, the Data Transparency Label^46^, and the Patient Privacy Rights Information Governance Label^47^, as well as efforts to make a cybersecurity “Energy Star”^48^. These types of labels create a visual representation for metrics like the type of data collected, retention period, update capabilities (e.g., automatic), and authentication methods, making it easier for less technical consumers to understand important features and gaps in a product.

### Mock visualizations

**Fig. 3:**
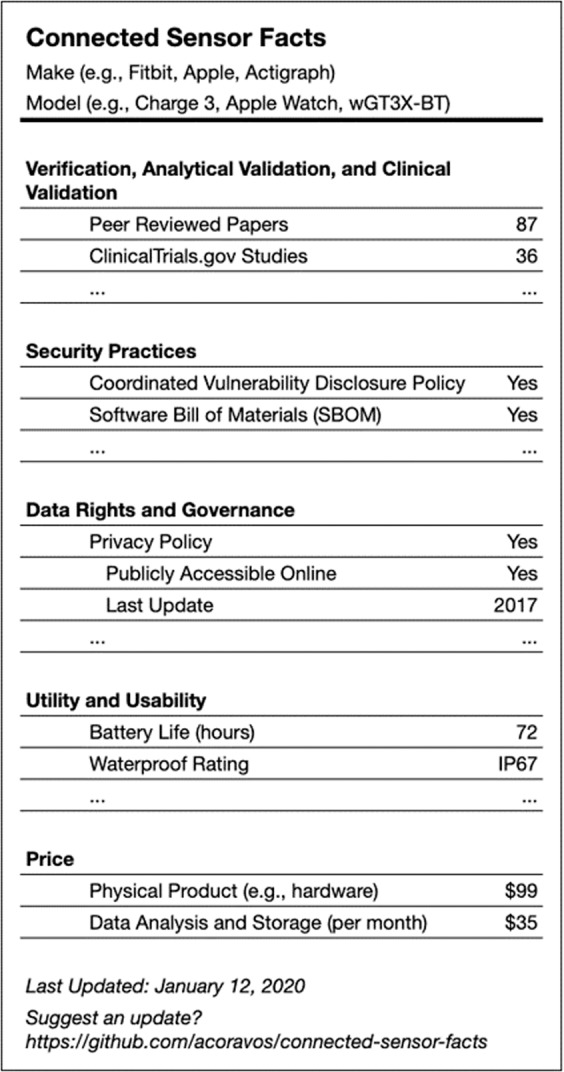
Connected sensor facts. This is an example of a nutrition label-type visualization that includes the key dimensions of the evaluative framework for connected sensor technologies, and metrics related to facts within each dimension. This approach permits a user to have a concise but comprehensive picture of a sensor’s appropriateness and fitness for use.

**Fig. 4:**
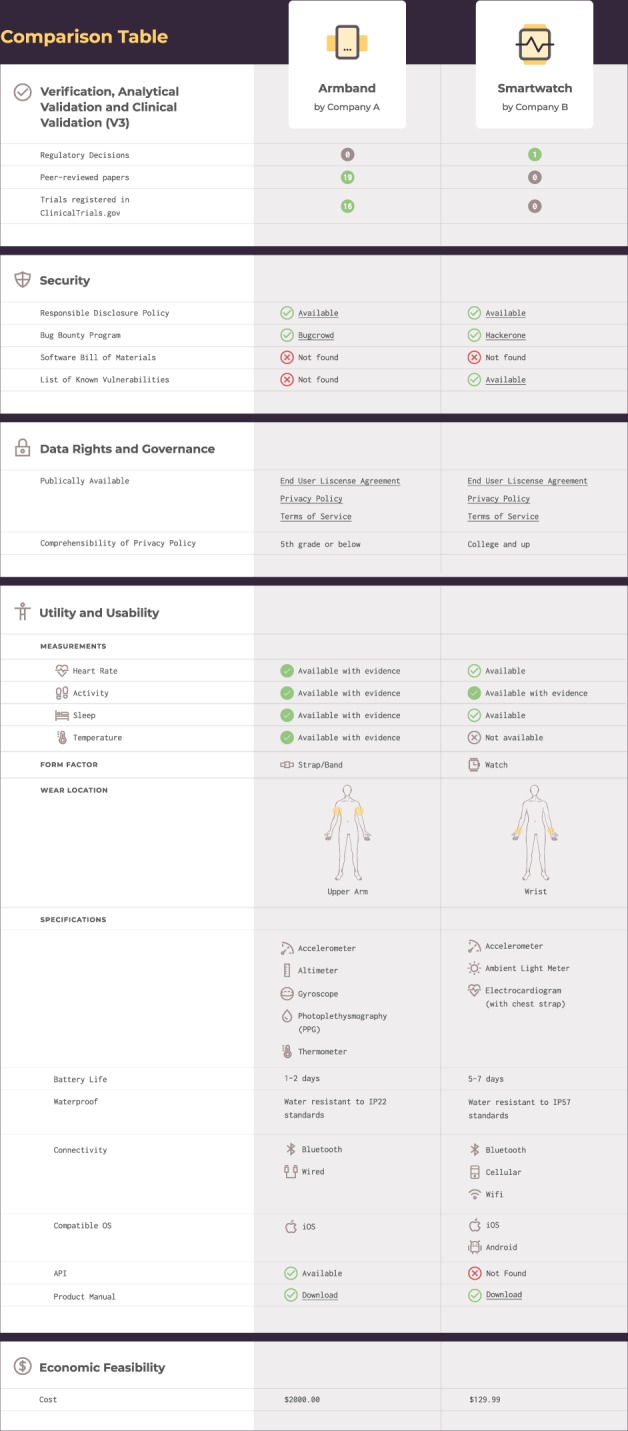
Connected sensor comparison table. This is an example of how sensors might be compared in a side by side visualization that incorporates dimensions from the proposed evaluative framework. In this illustrative visualization, additional detail and metrics are provided for the utility and usability dimension, in order to facilitate a rapid evaluation for appropriateness within an intended population.

We propose that a connected sensor technology label could be a useful piece of infrastructure for an evaluation framework, making it easier for decision-makers to understand critical aspects of the technology. The label format could demonstrate which connected sensor technologies are available that contain the proposed measurements, and whether each of five dimensions (V3, security practices, data rights and governance, utility and usability, and economic feasibility) meet minimal (pass/fail) or desired quality thresholds. It also displays additional quality, if any, for each dimension. A prototype is provided below, one that we hope will be the starting point for a broader industry discussion and ultimately, widespread implementation (Figs 3 and 4).

## Conclusions

The adoption of connected sensor technologies is not slowing down, and so it is of utmost importance to provide our community with improved and practical evaluation toolkits. Our hope is that the use of these toolkits will slow the adoption of substandard connected sensor technologies that lack evidence and/or harm individuals while accelerating the adoption of safe and effective ones. To achieve this vision, we need to define shared ontologies that better reflect the unique considerations of products in the digital era. Evaluation frameworks will need to be easy to understand and implement in order to shift the adoption curve at scale. Frameworks will also need to provide risk-benefit evaluations in a timely manner, as software organizations have the ability to make changes to connected products faster compared to other medical products (e.g., ability to ship software updates multiple times per day versus releasing a new drug or medical device every few years). Additional work should be done to ensure that our proposed framework appropriately meets the needs of stakeholders throughout the digital medicine ecosystem, and our proposed framework should be continually reevaluated to best capture benefits and risks when adopting evolving technologies. Communities, like the Digital Medicine Society (DiMe), will be essential to develop a professional home to serve software engineers, data scientists, clinicians, and other experts working in this field as new frameworks are developed^49^.

There are many ways to operationalize an improved evaluation framework for connected sensor technologies, and the optimal result will likely have a mix of actions ranging from regulatory, standards bodies, and communication tools like accessible labeling. The underlying principles we outlined for connected sensor technologies can be adapted for related digital medicine technologies^50^ like ePROs or interventional products like digital therapeutics (DTx)^14^, although neither are the immediate focus of this work. Our hope is that the community will build on the five dimensions described here developing more robust thresholds so more technologies are worthy of the trust society places in them.

### Reporting summary

Further information on research design is available in the Nature Research Reporting Summary linked to this article.

## Supplementary information

Reporting Summary

## Data Availability

No datasets were generated or analysed during the current study.
