# Publisher Correction: Modernizing and designing evaluation frameworks for connected sensor technologies in medicine

**DOI:** 10.1038/s41746-020-0263-1

**Published:** 2020-04-02

**Authors:** Andrea Coravos, Megan Doerr, Jennifer Goldsack, Christine Manta, Mark Shervey, Beau Woods, William A. Wood

**Affiliations:** 1 Elektra Labs, Boston, MA USA; 2 Harvard-MIT Center for Regulatory Science, Boston, MA USA; 3 Digital Medicine Society (DiMe), Boston, MA USA; 4 Biohacking Village, Washington, DC, USA; 5 Sage Bionetworks, Seattle, WA USA; 6 I Am The Cavalry, Washington, DC, USA; 7 Atlantic Council, Washington, DC, USA; 8 University of North Carolina Lineberger Comprehensive Cancer Center, Chapel Hill, NC USA

**Keywords:** Technology, Health policy

Correction to: *npj Digital Medicine* 10.1038/s41746-020-0237-3, published online 13 March 2020

The original HTML version of this Article was updated after publication following a technical error that resulted in the incorrect version of Figure 2 and Figure 4 appearing in the HTML version of the Article. This has now been corrected in the HTML version of the Article. The PDF version of the Article was correct at the time of publication.

